# Ileal Dysbiosis Is Associated with Increased Acoustic Startle in the 22q11.2 Microdeletion Mouse Model of Schizophrenia

**DOI:** 10.3390/nu15163631

**Published:** 2023-08-18

**Authors:** Julianne Ching Yang, Ryan Troutman, Heidi Buri, Arjun Gutta, Jamilla Situ, Ezinne Aja, Jonathan Patrick Jacobs

**Affiliations:** 1 The Vatche and Tamar Manoukian Division of Digestive Diseases, Department of Medicine, David Geffen School of Medicine, University of California Los Angeles, Los Angeles, CA 90095, USA; jcyang1617@g.ucla.edu (J.C.Y.); troutmanryan7@g.ucla.edu (R.T.); heidiburi@g.ucla.edu (H.B.); arjungutta@g.ucla.edu (A.G.); jamillasitu@gmail.com (J.S.); eaja@mednet.ucla.edu (E.A.); 2 Goodman-Luskin Microbiome Center, University of California Los Angeles, Los Angeles, CA 90095, USA; 3 Division of Gastroenterology, Hepatology and Parenteral Nutrition, VA Greater Los Angeles Healthcare System, Los Angeles, CA 90073, USA

**Keywords:** microbiome, schizophrenia, gut–brain axis, DiGeorge, 22q11.2, microdeletion

## Abstract

Recent studies involving transplantation of feces from schizophrenia (SCZ) patients and their healthy controls into germ-free mice have demonstrated that the gut microbiome plays a critical role in mediating SCZ-linked physiology and behavior. To date, only one animal model (a metabotropic glutamate receptor 5 knockout) of SCZ has been reported to recapitulate SCZ-linked gut dysbiosis. Since human 22q11.2 microdeletion syndrome is associated with increased risk of SCZ, we investigated whether the 22q11.2 microdeletion (“Q22”) mouse model of SCZ exhibits both SCZ-linked behaviors and intestinal dysbiosis. We demonstrated that Q22 mice display increased acoustic startle response and ileal (but not colonic) dysbiosis, which may be due to the role of the ileum as an intestinal region with high immune and neuroimmune activity. We additionally identified a negative correlation between the abundance of a *Streptococcus* species in the ilea of Q22 mice and their acoustic startle response, providing early evidence of a gut–brain relationship in these mice. Given the translational relevance of this mouse model, our work suggests that Q22 mice could have considerable utility in preclinical research probing the relationship between gut dysbiosis and the gut–brain axis in the pathogenesis of SCZ.

## 1. Introduction

Schizophrenia (SCZ) impacts approximately 24 million individuals, with a 1% prevalence rate worldwide [1]. Individuals with SCZ experience positive symptoms (e.g., hallucinations, delusions), negative symptoms (e.g., social withdrawal, difficulty keeping conversation), and cognitive deficits (e.g., attention, working memory, executive functioning) [1,2]. While existing treatments are capable of reducing symptoms, no cure exists.

The exact etiology of SCZ is not fully understood, as both genetic and environmental factors may be involved [3]. Encapsulating both genetic and environmental influences, the gut microbiome has recently been implicated in the pathogenesis and development of psychiatric disorders [4]. Nguyen et al. previously reported significant differences in microbiome beta-diversity between SCZ patients and their non-psychiatric controls, in addition to finding enrichment of members of the Lachnospiraceae family in SCZ patients [5]. Furthermore, a study combining fecal 16S rRNA gene sequencing with magnetic resonance imaging data from SCZ patients and matched controls identified significant correlations between microbiome alpha-diversity and brain region structure and functional indicators [6]. Microbiota transplantation studies in mouse models have been essential in establishing a causal role for the microbiota in mediating SCZ-linked outcomes. Zhu et al. reported that mice with transplanted microbiota from SCZ patients experienced SCZ-like symptoms such as hyperactivity, decreased anxiety, and depressive-like behaviors [7]. Another study found that mice with transplanted SCZ microbiota experienced impaired memory in addition to increased psychomotor activity [8].

These findings warrant additional investigation into the means by which the gut microbiota may influence SCZ. A bidirectional relationship has been shown to exist between the microbiota, gut, enteric nervous system, and central nervous system, also known as the microbiome–gut–brain axis [9]. Previous research has discovered that the gut microbiome can promote the expansion of CD4+ T cells and subsequent activation of immune signaling pathways [10]. Additionally, microbial metabolites such as short-chain fatty acids can regulate blood–brain barrier permeability and promote neuroinflammation that may affect neurological disease progression [11]. Since both systemic inflammation and neuroinflammation are characteristics of SCZ patients, it is plausible that the gut microbiome modulates SCZ through its effects on the immune response [12].

Identification of translationally relevant genetic animal models of SCZ that also show dysbiosis would advance our understanding of microbiome–gut–brain interactions in SCZ. Microdeletion in the 22q11.2 region is thought to be one of the strongest genetic predictors of SCZ [13]. Approximately 30% of individuals with deletion of chromosome 22q11.2 develop SCZ [14]. Mice modeling 22q11.2 deletion syndrome (hereafter referred to as Q22 mice) possess construct validity, since mice mimic both positive and negative symptoms of SCZ [15,16]. Q22 mice exhibit reduced sensorimotor gating, differences in social tolerance, and deficiencies in decision-making for long-term rewards and in tasks involving intact circuitry between the prefrontal cortex and hippocampus, such as the object-in-place assay [15,17]. To address whether Q22 mice may have additional mechanistic validity with respect to gut dysbiosis, we evaluated whether microbiome differences between Q22 and WT mice are associated with SCZ-like phenotypes.

## 2. Materials and Methods

### 2.1. Animals

Human 22q11.2 deletion [Df(h22q11)/+] (Nomenclature: C57BL/6-Del(16Dgcr2-Hira)1Tac) mice were ordered from Taconic Biosciences (New York, NY, USA) and bred for two generations with wild-type (WT) C57BL/6J mice to produce F2 generation male and female heterozygous deletion [Df(h22q11)/+] “Q22”and WT offspring, which were used in the study. Mice were housed on a 12/12 h light/dark cycle in specific pathogen-free conditions with autoclaved bedding and were given water and irradiated food (PicoLab Rodent Diet 5053, Fort Worth, TX, USA) ad libitum. The open-field (Q22 *n* = 7, WT *n* = 13), object location memory (Q22 *n* = 5, WT *n* = 5), and acoustic startle-prepulse inhibition (PPI) (Q22 *n* = 11, WT *n* = 15) assays were performed at weeks 10, 12, and 16, respectively. Mice were euthanatized on the day following the startle-PPI assay. Intestinal luminal content expressed from the ileum (as defined by the distal third of the small intestine) and the colon (excluding the cecum) were utilized for 16S v4 rRNA gene sequencing on Illumina MiSeq (Q22 *n* = 13, WT *n* = 15). All animal experiments were approved by the University of California Los Angeles Animal Research Committee, University of California Los Angeles (approval code: 2003-014 approval date: 3 November 2003).

### 2.2. Handling

To minimize the effects of experimenter handling stress for object-in-place and startle-PPI behaviors, we handled mice for 7 days, with 1 day of rest on the 6th day. The mice were increasingly handled over the course of the days, where handling was limited at the beginning (e.g., touching the mice, grabbing them by the tail) and increased by the end of the 7 days (e.g., holding them in the hands and petting each mouse individually). Following handling, the mice were habituated in the testing room for 1 h without seeing the open-field box and then added to the testing arena (box) to habituate for 12 min over the course of 2 days. The protocol was adapted from the work of Manuel F. Lopez Aranda and Alcino Silva, UCLA.

### 2.3. Acoustic Startle Response and Prepulse Inhibition of Startle

The startle-PPI test was used to assess the prepulse inhibition (PPI) in the acoustic startle response. Gloves were changed between home cages, and the startle-PPI apparatus (SR-LAB-Startle Response System, San Diego Instruments, San Diego, CA, USA) was cleaned with 50% Windex and allowed to dry for 5 min between mice. The apparatus was calibrated at the beginning before the mice were placed. Following the restraint of mice within the central enclosure of the apparatus, the mice acclimated to a constant background white noise for 5 min. Following this, mice underwent six 120 db startle presentations. Next, pseudo-randomized prepulse inhibition phases occurred for 17 min in trial combinations of prepulse (70, 75 or 80 db) + pulse (120 db) and concluded with six 120 db startle presentations. The data were exported from the SR-Lab program and analyzed in R. To analyze the % prepulse inhibition, the average Vmax values for each animal and type of trial were averaged. We calculated the percent inhibition for each db prepulse using the following formula (the 70 db formula is shown here as an example): 100 − 100(average PPI 70 db/average 120 db middle 10 trials) = percent inhibition from 70 db prepulse. Differences in acoustic startle response and % prepulse inhibition between Q22 and WT mice were assessed with the Wilcoxon rank-sum test.

### 2.4. Open-Field Test

To explore anxiety-like behaviors and general locomotor activity, the open-field assay was performed in four 30 cm × 30 cm quadrants. Mice were habituated for 30 min in the testing room before testing began. Gloves were changed between every home cage. Mice were individually placed into one of four quadrants and recorded for 10 min. Videos were analyzed using AnyMaze software (v. 7.0). A 6 cm grid was centered over each quadrant. A 3 × 3 (18 × 18 cm) inner square was used to evaluate the time that the mice spent in the center vs. the periphery (18 × 18 cm). The middle of the mouse’s body was used to track its location in the quadrant. The Wilcoxon rank-sum test was used to assess whether there were significant differences in total distance traveled (cm) or time in the center (seconds) by genotype (Q22 or WT).

### 2.5. Object-in-Place Test

To assess mice’s cognition, specifically spatial memory and discrimination, the object-in-place test was conducted. The box was set up to include four 30 cm × 30 cm quadrants. Mice were habituated to the testing room 1 h prior to the assay. Gloves were changed between every home cage. The behavioral testing box was set up so that four objects were placed in each respective corner (6 cm from each wall), with each object being unique. The objects used were a glass bottle with clear liquid, a glass bottle with blue liquid, a brown bottle with a black cap, and a large glass bottle with clear liquid. Mice were placed into one of four quadrants that contained 4 novel objects and were recorded for 10 min. The mice were taken out of the box, the box was cleaned with 70% ethanol, and two objects were switched. The switched objects were the same for each mouse tested. The mice were reintroduced after 5 min and recorded for 3 min. The discrimination ratio was calculated to determine how long the mouse spent with the new object and old objects as a function of memory and cognition. Videos were analyzed using AnyMaze software (v. 7.0). A tightly drawn circle with a 2 cm radius from the center of the object and an orientation cone was employed to measure the time spent investigating the object. Investigation time was only counted when the mouse’s head was inside the circle and the object was within the 90-degree orientation cone. The protocol for this assay was adapted from the work of Tripathi et al., 2020 [15]. Differences in discrimination ratio between Q22 and WT mice were assessed for significance with Wilcoxon’s rank-sum test.

### 2.6. 16S V4 rRNA Gene Sequencing

DNA was extracted from ileal and colonic luminal content using the Qiagen DNeasy PowerSoil Pro Kit (Cat. no. 47014) according to the manufacturer’s instructions. Amplification of the V4 region of the 16S rRNA gene and barcoding were performed as previously described [18]. Products were pooled in equal amounts, and the resulting library was sequenced on an Illumina MiSeq using the 2 × 250 bp v2 kit to a mean depth of 20,653 reads. A DNA extraction blank and no template control negative controls were included in the assays.

### 2.7. Sequencing Data Preprocessing

Raw amplicon reads underwent quality control, merging of forward and reverse reads, and denoising with the DADA2 package in R, resulting in an amplicon sequence variant (ASV) × samples count table [19]. Taxonomy assignment of representative sequences was achieved with the naive Bayesian classifier implemented in the assignTaxonomy DADA2 function using the Silva v138.1 database provided on Zenodo (https://zenodo.org/record/4587955, accessed on 14 July 2023) [19,20,21].

### 2.8. Data Analysis

The count table was split into subsets of ileum and colon samples using QIIME2 (v. 2022.2). For analysis of microbiome alpha-diversity, the subsets were first rarefied (to 15,940 and 3370 reads for the ileum and colon subsets, respectively) and then utilized to calculate the number of unique ASVs for species richness or calculate Shannon’s entropy for species evenness and richness using QIIME2 (v. 2022.2). All other data visualization, statistics, and analysis were carried out in R (v. 4.2.0). For assessment of microbiome beta-diversity, the ileum and colon count tables were first prevalence-filtered to retain ASVs present in at least 15% of samples. The vegan package (v. 2.6-4) was used to calculate a robust Aitchison sample dissimilarity matrix of the filtered count tables and PERMANOVA of sample dissimilarities to estimate R^2^ and *p*-values associated with genotype after accounting for litter and sex covariates. Per-ASV association testing with genotype was accomplished through fitting linear mixed-effects models implemented in the package Maaslin2 (v. 1.12.0) on log-transformed, total-sum-scaling-normalized, prevalence-filtered count tables with genotype and sex as fixed effects and litter as a random effect. An ASV was considered to be statistically significant if the q-value (Benjamini–Hochberg-corrected *p*-value) fell below a value of 0.05. To assess whether there were significant correlations between the abundance of the significantly differentially abundant *Streptococcus* ASV and acoustic startle response, Spearman correlation tests implemented in the R package stats (v. 4.2.0) of startle VMax and the relative abundances of the *Streptococcus* ASV were performed.

Data visualization of differences by genotype in alpha-diversity indices, behavioral data metrics, and relative abundance of the *Streptococcus* ASV was performed using boxplots implemented in the package ggplot2 (v. 3.4.2). For comparisons of alpha-diversity and behavioral metrics by genotype, the Wilcoxon rank-sum test implemented in the stats package (v. 4.2.0) was used. Differences were considered significant if the *p*-value fell below 0.05. The data input files and R scripts associated with this project can be accessed on https://github.com/julianneyang/q22, accessed on 20 July 2023.

## 3. Results

**Figure 1:**
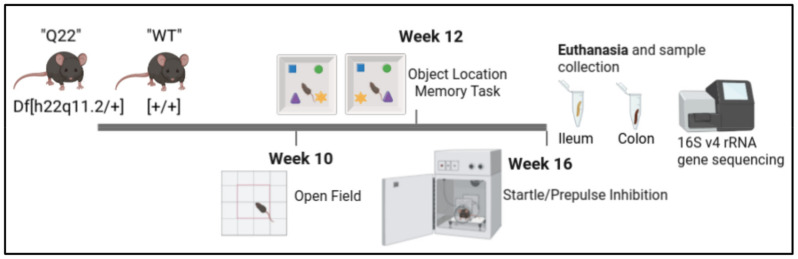
Study design: Q22 mice and their WT littermate controls underwent behavioral testing at 10, 12, and 16 weeks of age. At 16 weeks, the mice were euthanatized and their ileum and colon luminal content samples were collected. DNA extracted from ilea and colons underwent V4 amplicon sequencing of the 16S rRNA gene.

We performed behavioral testing and intestinal microbiome sequencing of Q22 and WT mice to elucidate the microbiome differences between Q22 and WT mice and address whether the Q22-linked microbiome is associated with SCZ-like phenotypes (Figure 1). Because Q22 mice may exhibit certain SCZ phenotypes in an age-dependent manner, we performed the open-field test at week 10, the object-in-place test at week 12, and the startle PPI at week 16 [17].

### 3.1. Q22 Mice Exhibit Sensorimotor Gating Deficits Compared to WT Counterparts, but Not Abnormalities in General Locomotor Activity, Anxiety-like Behavior, or Cognition

Acoustic startle response and prepulse inhibition of acoustic startle (PPI) are the most reliable and widely reported SCZ-linked behaviors in animal models [22]. The acoustic startle response is the immediate and robust reflexive reaction to an intense sensory stimulus, whereas sensory gating is the phenomenon whereby the brain exhibits reduced responses to repeated stimuli [23]. Individuals with schizophrenia exhibit impaired sensorimotor processing [24]. In the startle PPI assay, mice are presented with a weak stimulus (known as the prepulse) shortly before a strong, startling stimulus (known as the pulse or the startle). When presented with the prepulse, mice with normal gating will have a reduced response to the startling stimulus. Mice with abnormal sensorimotor processing may display both increased acoustic startle and reduced prepulse inhibition [25].

**Figure 2:**
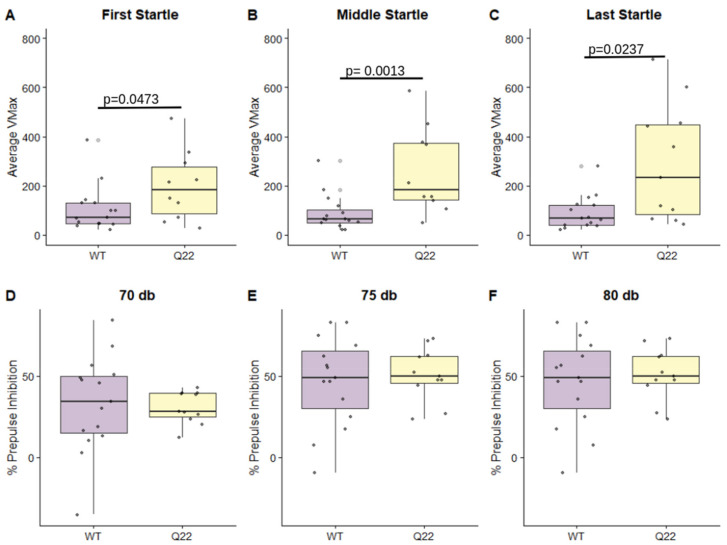
Q22 mice exhibit increased acoustic startle response but not prepulse inhibition of acoustic startle compared to WT mice: Boxplots comparing the distributions of average Vmax for WT and Q22 mice for the first set (**A**), the middle set (**B**), and the last set of startle presentations (**C**). Boxplots comparing the % prepulse inhibition at 70 db (**D**), 75 db (**E**), and 80 db prepulses (**F**) for WT and Q22 mice. Significance was assessed with the Wilcoxon rank-sum test.

We observed that Q22 mice had significantly increased average Vmax (peak velocity during startle response) in the first set of startle presentations, middle startle presentations, and last startle presentations compared to their WT counterparts, suggesting an abnormal startle response in Q22 mice (*p* < 0.05) (Figure 2A–C). However, we did not observe significant differences in % prepulse inhibition at 70 db, 75 db, or 80 db prepulse levels between WT and Q22 mice (Figure 2E,F).

**Figure 3:**
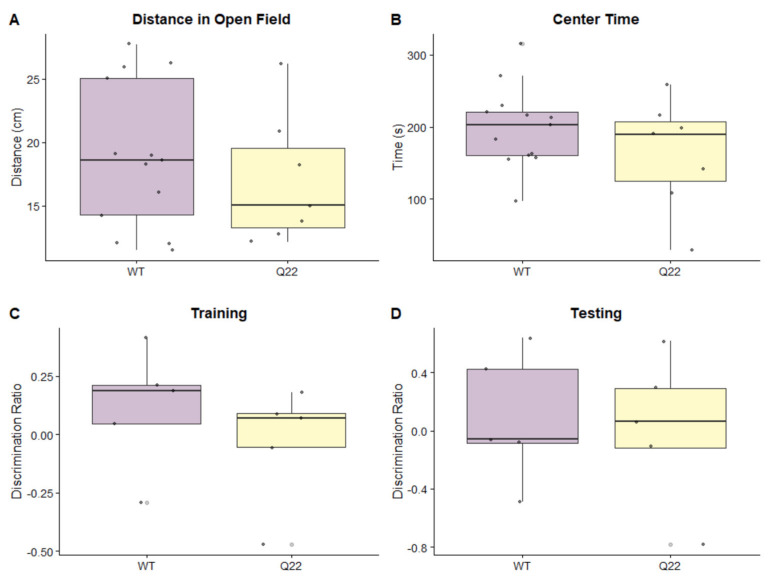
Q22 mice do not exhibit abnormalities in the open-field or object-in-place tasks: Boxplots comparing (**A**) the total distance traveled in centimeters (cm) and (**B**) the time the mice spent in the center (in seconds) between WT and Q22 mice during the 10 min open-field assay. Boxplots showing the discrimination ratio for (**C**) the training phase and (**D**) the testing phase for WT and Q22 mice in the object-in-place task, with 0.00 representing no preference for either set of objects.

Since others have reported that genetic mouse models of schizophrenia (e.g., Disc1) exhibit alterations in anxiety-like behaviors and locomotor activity compared to their wild-type counterparts, we also assessed whether these behaviors were present in Q22 mice [26,27,28]. The open-field test is a validated and commonly used assay to assess anxiety-like and locomotor activities in SCZ mouse models [29]. The anxiety and locomotor phenotypes can manifest in various measures, including exploratory behavior and distance traveled. Mice with increased anxiety-like behavior prefer to stay close to the walls, manifesting in reduced center time [29]. We observed no significant differences in distances traveled between WT and Q22 KO mice, or in time spent in the center vs. the periphery (Figure 3A,B).

Q22 mice have also been reported to exhibit alterations in spatial memory and discrimination, which can be assessed through the object-in-place task [15]. The discrimination ratio was calculated as the difference in time spent exploring the objects that changed location compared to the time spent exploring the objects that did not change location, which was expected to increase in the testing phase. We observed no significant differences between Q22 and WT mice in the object-in-place assay in the training or testing phases (Figure 3C,D).

### 3.2. The Q22 Genotype Is Associated with Changes in the Ileal Microbiome

**Figure 4:**
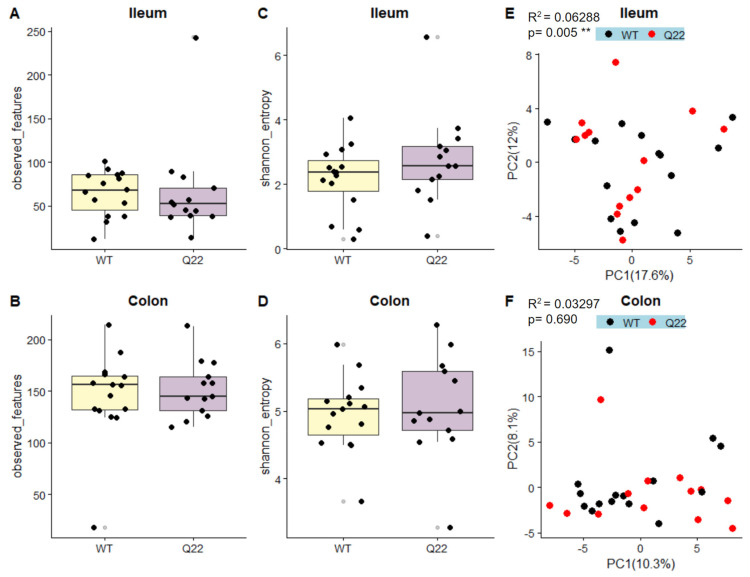
Q22 mice exhibit differences in ileal microbiome diversity: Boxplots depicting species richness as assessed through the total number of observed ASVs (observed features) in Q22 and WT mice for the ileum (**A**) and colon (**B**) samples. Species evenness and richness of ileum (**C**) and colon (**D**) samples as assessed through Shannon’s entropy are shown for Q22 and WT mice. Principal coordinate analysis (PCoA) plots of robust Aitchison distances illustrating the unsupervised clustering of Q22 (red) and WT (black) ileal (**E**) and colonic (**F**) samples. PERMANOVA R^2^ and *p*-values (** *p* < 0.01) associated with genotype (WT vs. Q22) are reported in the upper left of each PCoA plot.

Next, to assess whether differences in the microbiome exist between Q22 and WT mice, we performed 16S V4 rRNA gene sequencing of both ileum and colon luminal contents (Figure 1). We selected the colon for sequencing, given the use of fecal samples in previous studies comparing the microbiomes of schizophrenia patients to healthy controls [30]. However, since patients with 22q11 microdeletion typically present with T-cell deficiency, we speculated that the effect of 22q11 microdeletion may be most apparent in the ileum, an intestinal region with high immune cell activity [31]. Though there were no differences in species richness as assessed through the number of ASVs or in evenness and richness as assessed by Shannon’s entropy, genotype was significantly associated with ileal but not colonic global microbiome composition, as assessed through PERMANOVA of robust Aitchison dissimilarities (Figure 4).

### 3.3. A *Streptococcus* ASV Distinguishes Q22 Mice from WT Mice and Is Significantly Correlated with Acoustic Startle Reflex

**Figure 5:**
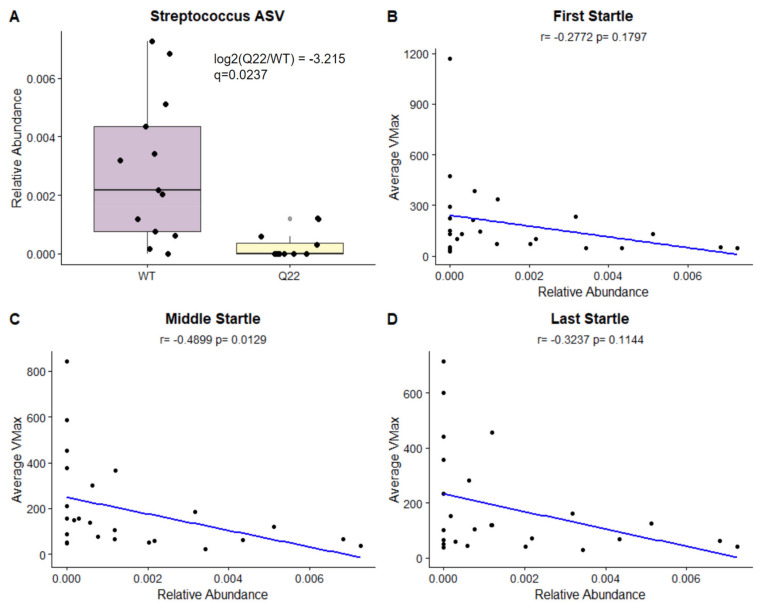
A *Streptococcus* ASV is significantly reduced in KO compared to WT mice and is negatively correlated with acoustic startle response: (**A**) Boxplots illustrating the within-sample relative abundance of a Q22-depleted ASV belonging to the genus *Streptococcus*. The log2 fold-change of this ASV and the q-value of the association in MaAsLin2 models are shown. (**B**–**D**) Scatterplots with trend lines depicting the relationship between the average VMax and relative abundance of the ASV for the first set (**B**), middle set (**C**), and last set (**D**) of startle presentations. The correlation coefficients and *p*-values are shown at the top of the scatterplots.

We then performed differential abundance testing of amplicon sequence variants (ASVs), which roughly correspond to species, by fitting the count data to linear mixed-effects models implemented in MaAsLin2. This resulted in identification of an ASV belonging to the genus *Streptococcus* that was significantly reduced in Q22 compared to WT mice (Figure 5A). Spearman’s correlation tests of the average VMax during the first, middle, or last set of startle presentations with *Streptococcus* ASV abundance revealed a significant negative association of the *Streptococcus* ASV with the average VMax during the middle set of startle presentations (Figure 5B,D). These results suggest that a reduction in this particular *Streptococcus* ASV in Q22 mice is linked to the sensorimotor processing deficits in Q22 mice.

## 4. Discussion

SCZ mouse models with both face validity (recapitulating phenotypes with disorder-defining symptoms) and mechanistic validity (recapitulating a biological mechanism associated with the disorder) are scarce, which complicates preclinical research into the biological mechanisms involved in this disorder [32]. To date, only one other study has reported gut dysbiosis in a genetic SCZ mouse model—a metabotropic glutamate receptor-5-knockout mouse model—limiting options for preclinical investigation into the role of dysbiosis in SCZ [33].

Here, we demonstrate for the first time that the Q22 mouse model has altered microbial composition, establishing that it has mechanistic validity with respect to gut dysbiosis observed in SCZ. Consistent with findings in human gut microbiome studies of SCZ patients and their healthy controls, we did not observe any differences in alpha-diversity but observed significant Q22-linked differences in beta-diversity [6,34]. Additionally, we identified a *Streptococcus* ASV that was 8.5 times less abundant in Q22 mice compared to their WT controls. The genus *Streptococcus* has previously been identified as significantly differentially abundant between SCZ patients and their healthy controls, although the direction of the association is inconsistent between studies [34,35]. However, the abundance of this ASV was negatively correlated with the acoustic startle response across all mice, suggesting that the reduced abundance of this microbe is indeed related to the SCZ phenotype. This preliminary evidence of a gut–brain relationship may mirror findings in humans, where symptom severity score was negatively correlated with the abundances of several microbes and their metabolites, including *Streptococcus salivarius* [36].

It is important to note that the microbial changes that we report here may be (1) driven by a direct effect of the 22q11.2 microdeletion on the microbiota, leading to gut dysbiosis serving as an intermediate effector between genetic susceptibility and an SCZ-like outcome; (2) mediated by secondary effects of the 22q11.2 microdeletion on the brain, in which case gut dysbiosis reflects the disease state; or (3) a combination of both (1) and (2). Supporting the first idea, evidence from two studies of humanized germ-free mice suggest that gut dysbiosis could contribute to SCZ phenotypes, although one study highlights modulation of kynurenine–tryptophan metabolism by SCZ-linked microbiota, while the other emphasizes microbial modulation of the glutamine–GABA cycle as a possible mechanism [7,8]. However, a similar transplantation study involving humanization of WT germ-free mice with the microbiome of SCZ patients with 22q11.2 deletion, patients with 22q11.2 deletion but not SCZ, or their healthy controls would still be required to assess whether the 22q11.2-linked microbiota can drive SCZ. Additionally, a strength of utilizing the Q22 mouse model as a model of SCZ is that facility-specific effects on disease may actually be physiologically relevant. For example, Forster et al. recently found that the gut microbiome variability between institutions could affect the disease outcome in the widely used dextran sulfate sodium model of colitis [37]. Along the same lines, differences in housing conditions across studies can affect the gut microbiome and lead to differences in behavioral outcomes [38]. A facility-specific housing condition that leads to the loss of SCZ-like phenotypes in the Q22 mice would perhaps lend credence to the idea that microbes drive SCZ in Q22 mice, or if enough investigators combine behavioral phenotyping with gut microbiome analysis in their studies across several facilities with near-identical housing conditions, a consistent Q22-driven microbiome signature may emerge.

Though no study has investigated gastrointestinal function in Q22 mice, in a study of 206 patients with 22q11.2 deletion syndrome, 91% experienced gastrointestinal symptoms, but only 3.5% and 1.5% exhibited congenital gastrointestinal malformations and gastrointestinal autoimmune disorders, respectively [39]. Chronic gastrointestinal symptoms were also significantly associated with psychiatric comorbidities [39]. Taken together, the observation in human 22q11.2 deletion syndrome patients suggests that the effect of 22q11.2 microdeletion on the gut is a functional and not a structural effect, supporting the possibility that gut dysbiosis may be secondary to the effects of the microdeletion on the brain.

Behavioral phenotypes in the Q22 mice partially reproduced prior findings. We observed increased acoustic startle response in the Q22 model, but in contrast to previously published studies we did not observe reduced % prepulse inhibition in Q22 mice [15,17]. This may have been confounded by the robust increased startle response at baseline or “first startle”—meaning prior to any prepulse + pulse trial—requiring a larger sample size to elucidate the effect on % prepulse inhibition [40]. In general, small sample sizes, particularly for the open-field and object-in-place tasks, were a limitation in this study.

One key distinction between our study and previously published gut microbiome studies on human SCZ patients is the sample type used for microbiome assessment. While previous human gut microbiome studies utilized feces, we selected ileal content in addition to fecal content for sequencing in the Q22 mice. Interestingly, observed differences in the gut microbiome between Q22 and WT mice were localized to the ileum, while we did not identify microbiome differences in the colon. We speculate that this may be due to the ileum being an intestinal region with high immune and potentially neuroimmune activity, though additional small-intestinal microbiome transplantation experiments investigating whether the Q22-linked small-intestinal microbiome drives immune and neuroimmune activity in Q22 mice are necessary [41,42].

Based on our findings, the Q22 mouse model is a strong candidate for preclinical research investigating the role of the gut microbiome in schizophrenia, as it recapitulates both SCZ-linked behavior and gut dysbiosis. Additional experimentation identifying whether and how the Q22-linked microbiome mediates SCZ-like phenotypes could lead to future opportunities to develop novel probiotics and prebiotics that modulate the SCZ-associated microbiome.

## Data Availability

The input files used for analysis and the accompanying R scripts can be found at https://github.com/julianneyang/q22, accessed on 20 July 2023. Raw sequencing data are publicly accessible on NCBI with accession code PRJNA997603.
